# Anesthesia for thymectomy

**DOI:** 10.4103/1658-354X.76465

**Published:** 2011

**Authors:** Abdelazeem El-Dawlatly

**Affiliations:** *Professor of Anaesthesia, College of Medicine King Saud University, Riyadh, KSA E-mail: dawlatly@ksu.edu.sa*

Anesthesia for thymectomy in patients with myasthenia gravis is challenging. Myasthenia gravis is an autoimmune neuromuscular disease affecting the postsynaptic acetylcholine receptors that are blocked by specific antibodies, with resultant muscle weakness and fatigue. Anesthesia for thymectomy can be classified into: muscle relaxant or nonmuscle relaxant techniques. Our description of the nonmuscle relaxant technique retuned back to 1994, when it was first described.[1] The technique included insertion of a thoracic epidural catheter in an awake patient followed by induction of anesthesia using propofol and fentanyl. Tracheal intubation was facilitated with spraying the vocal cords with 3 ml of 4% xylocaine and maintenance of anesthesia was achieved with oxygen in air and 10–15 ml of 2% propofol as continuous i.v. infusion drip. Our nonmuscle relaxant technique has got wide acceptance and was verified in many subsequent studies worldwide.[2,3] In this issue of Saudi J Anesthesia, Stephenson *et al*. further described a nonmuscle relaxant technique for trans-sternal thymectomy in patients with juvenile myasthenia gravis with satisfactory outcome results.[4] We believe that the use of a nonmuscle relaxant anesthetic technique for trans-sternal thymectomy provides excellent intra- and postoperative conditions. Recently, thoracoscopic thymectomy has got wide acceptance among surgeons. In that regard, we have described a modified anesthetic technique that we believe is suitable for thoracoscopic thymectomy with excellent outcome results.[5]
